# Investigation of the In Vitro Antioxidant Potential Of Polyphenolic-Rich Extract of *Artocarpus heterophyllus* Lam Stem Bark and Its Antidiabetic Activity In Streptozotocin-Induced Diabetic Rats

**DOI:** 10.1177/2515690X20916123

**Published:** 2020-05-18

**Authors:** Basiru Olaitan Ajiboye, Oluwafemi Adeleke Ojo, Babatunji Emmanuel Oyinloye, Mary Abiola Okesola, Adeyonu Oluwatosin, Aline Augusti Boligon, Abidemi Paul Kappo

**Affiliations:** 1 Afe Babalola University, Ado-Ekiti, Ekiti State, Nigeria; 2 Landmark University, Omu-Aran, Kwara State, Nigeria; 3 University of Zululand, KwaDlangezwa, South Africa; 4 Covenant University, Ota, Ogun State, Nigeria; 5 Federal University of Santa Maria, Santa Maria, Brazil; * Current address: Molecular Biophysics and Structural Biology Group, Department of Biochemistry, Faculty of Science, University of Johannesburg, Johannesburg, South Africa.

**Keywords:** antioxidant, hexokinase, glycogen, pancreatic β-cell scores, lipid peroxidation

## Abstract

*Artocarpus heterophyllus* Lam (Moraceae) stem bark has been used locally in managing diabetes mellitus with sparse scientific information. This study investigates the in vitro antioxidant potential of polyphenolic-rich extract of *A heterophyllus* stem bark as well as its antidiabetic activity in streptozotocin-induced diabetic rats. Fifty male Wistar rats were used with the induction of diabetes by a single intraperitoneal injection of streptozotocin (45 mg/kg body weight) and were orally administered 400 mg/kg free and bound phenols of *A heterophyllus* stem bark. The animals were sacrificed on the 28th day of the experiment using the cervical dislocation method; antihyperglycemia and anti-inflammatory parameters were subsequently assessed. The polyphenolic extracts demonstrated antioxidant potentials (such as hydrogen peroxide and diphenyl-1-picrylhydrazyl), as well as strong inhibitory activity against amylase and glucosidase. There was a significant (*P* < .05) increase in glycogen, insulin concentration, pancreatic β-cell scores (HOMA-β), antioxidant enzymes and hexokinase activities, as well as glucose transporter concentration in diabetic animals administered the extracts and metformin. Also, a significant (*P* < .05) reduction in fasting blood glucose, lipid peroxidation, glucose-6-phosphatase, and all anti-inflammatory parameters were observed in diabetic rats administered the extracts and metformin. The extracts demonstrated antidiabetic potential, which may be useful in the management of diabetes mellitus

Diabetes mellitus is a metabolic disease characterized by hyperglycemia and impaired glucose, lipid, and protein metabolism.^1,2^ This is due to insulin deficiency, leading to abnormal high blood glucose levels called hyperglycemia.^3^ The resulting hyperglycemia from defects in insulin action or insulin production may lead to a number of complications, which may include changes in biochemical parameters such as the formation of advanced glycation end-products enhanced by expression of pro-inflammatory cytokine genes.^4,5^ Excessive reactive oxygen species (ROS) production in diabetes mellitus patients due to a reduction in antioxidant enzymes can lead to tissue injury or apoptosis.^6^ Both oxidative stress and inflammation play chief roles in the development of insulin resistance, dyslipidemia, β-cell dysfunction, liver malfunction, and nephropathy among other ailments.^6,7^

Persistent high blood glucose levels can trigger a reduction in liver glycogen concentration and glycolysis enzyme activities; this may also result in an abnormal increase in gluconeogenesis enzyme activities due to deficiency in insulin secretion.^8^ In addition, hyperglycemia increases inflammatory markers such as tumor necrosis factor (TNF)-α, interleukins (eg. IL-1, IL-6, etc), and nuclear factor-κB, among others.^9,10^ Overproduction of pro-inflammatory cytokines enhances inflammatory stress in diabetes mellitus patients leading to various complications. According to the World Health Organization,^11^ more than 422 million adults worldwide are suffering from this complex multifactorial disease. Several conventional drugs like metformin and glimepiride, among others, have been used to manage diabetes mellitus worldwide but they are characterized by severe side effects such as vomiting, nausea, hypoglycemia, abnormal weight gain, and renal impairments. Additionally, lack of accessibility (especially in the rural areas) and affordability, particularly with the current economic meltdown globally,^12^ has necessitated the study and use of unconventional sources of antidiabetic drugs, without or with reduced adverse effects.^13^ Different parts of *A heterophyllus* such as the stem, bark, leaves, roots, and fruits have been documented as effective in the management of diabetes mellitus with no side effects.^14^

Ajiboye et al^14^ reported that *A heterophyllus* Lam (jack fruit) belongs to the Moraceae family, which grows in tropical climates. It is a rich source of carbohydrates, minerals, dietary fiber, and vitamins.^15^ Locally, this plant has been used in the management of not only diabetes mellitus but also for hypertension, hepatitis infections, and other ailments in some parts of Nigeria and other African countries.^14^ Some reports have been documented on the ethanol extract of this plant in the management of diabetes mellitus by Ajiboye et al^14,16^ but with sparse or no information on the antihyperglycemic and anti-inflammatory effects of the polyphenolic extract of the plant. Hence, the focus of this study is to investigate the in vitro antioxidant potential of polyphenolic-rich extract of *Artocarpus heterophyllus* Lam stem bark as well as its antidiabetic activity in streptozotocin-induced diabetic rats.

## Materials and Methods

### Sample Collection and Authentication

The freshly peeled stem bark of *Artocarpus heterophyllus* was obtained on the 10th of September 2015 at a farm in Ibadan, Oyo State, Nigeria. The bark was identified and authenticated by a senior taxonomist (Mr Omotayo) at the Department of Plant Science, Ekiti State University, Ado-Ekiti, Nigeria, with a voucher specimen number UHAE 119.

### Sample Preparation

The stem bark of *Artocarpus heterophyllus* was air-dried at room temperature (25°C) for 4 weeks to constant weight and then grounded into a fine powder using an electric blender. This was then stored at room temperature in an air-tight container.^14^

### Chemicals

All chemicals such as acetone, sodium hydroxide, hydrochloric acid, ethylacetate, sodium phosphate, potassium ferricyanide, 1,1-diphenyl-2-picryl-hydrazil, gallic acid, hydrogen peroxide, ascorbic acid, dinitrosalicylic acid, and p-nitrophenylglucopyranoside were bought from Sigma-Aldrich (St Louis, MO), while all the assay kits used were procured from (Randox Laboratories, Antrim, UK.

### Extraction of Free Phenol

Briefly, 10 g of *Artocarpus heterophyllus* stem bark (in powder form) was extracted using 80% acetone (1:5 w/v) for 72 hours, then filtered with the aid of Whatman No. 1 filter paper. Thereafter, the filtrate was evaporated to dryness using a rotary evaporator under vacuum at 45°C. This extract was then stored at −4°C for subsequent analyses. Also, the residue obtained during the filtration process was kept for the extraction of bound phenolics.^17^

### Extraction of Bound Phenol

The obtained residue from the above extraction was flushed with nitrogen and hydrolyzed with 20 mL of 4 M NaOH solution at room temperature for 1 hour with the aid of a shaker. Then, the pH of the mixture was adjusted to 2 using concentrated HCl and the bound phytochemicals were extracted with ethylacetate (6 times). Then the acquired ethylacetate fractions were evaporated to dryness using a rotary evaporator at 45°C.^17^

### Experimental Animals

A total of 50 male Wistar rats (aged 6 to 8 weeks) weighing between 150 and 170 g, obtained from the Animal Holding Units of Afe Babalola University, Ado-Ekiti, were used for this study. The animals were kept in clean plastic cages and a well-ventilated house. All animals were allowed free access to Afe Babalola University Animal feed (commercial feed) and water for a week before the commencement of the experiment as well as throughout the experimental period.

### Determination of Ferric Reducing Antioxidant Potential (FRAP)

The method described by Pulido et al^18^ was used in this determination. Briefly, 2.5 mL of the extract was mixed with 2.5 mL 200 mM sodium phosphate buffer (pH 6.6) and 2.5 mL of 1% potassium ferricyanide. The mixture was incubated at 50°C for 20 minutes and 2.5 mL of 10% trichloroacetic acid (TCA) was added to the mixture. Thereafter, it was centrifuged at 650*g* for 10 minutes, and 5 mL of the supernatant was mixed with equal volumes of distilled water and 1 mL 0.1% ferric chloride and the absorbance was read at 700 nm. The FRAP was calculated and expressed as gallic acid equivalent.

### Determination of 1,1-Diphenyl-2-Picryl-Hydrazil (DPPH) Radical Scavenging Ability

A solution of DPPH (0.135 mmol/L) in methanol was prepared and 1 mL of the solution was added to 3 mL of the extract suspension in water at different concentrations. The mixture was incubated for 30 minutes and absorbance was measured at 517 nm using an AJ-1C03 spectrophotometer. Gallic acid was used as a reference.^19^

### Determination of Hydrogen Peroxide Scavenging

The phenolic extract was dissolved in 0.1 nM phosphate buffer (pH 7.4) at various concentrations and mixed with 600 µL of hydrogen peroxide solution. Ascorbic acid was used as the reference compound. The absorbance values of the reaction mixture were read at 230 nm after 10 minutes.^20^

### Determination of α-Amylase Inhibitory Activity

Different concentrations of 250 µL volumes of the extract were incubated at 25°C for 10 minutes with 500 µL of hog pancreatic amylase (2 U/mL) in 100 mmol/L phosphate buffer (pH 6.8). After this, 250 µL of 1% starch dissolved in 100 mmol/L phosphate buffer (pH 6.8) was added to the mixture and incubated at 25°C for 10 minutes, followed by the addition of 1 mL of dinitrosalicylic acid (color reagent), which was then boiled for 10 minutes. The absorbance was measured at 540 nm. The inhibitory activity was expressed as a percentage of the control sample without inhibitors.^21^

### Determination of α-Glucosidase Inhibitory Activity

Substrate solution of p-nitrophenyl glucopyranoside (pNPG) was prepared in 20 mM phosphate buffer (pH 6.9). A 100 µL sample of α-glucosidase was pre-incubated with 50 µL of the different concentrations of the extracts for 10 minutes. Afterward, 50 µL of 3.0 mM (pNPG) as a substrate, dissolved in 20 mM phosphate buffer (pH 6.9), was added to start the reaction. The reaction mixture was incubated at 37°C for 20 minutes and stopped by adding 2 mL of 0.1 M Na_2_CO_3_. The α-glucosidase activity was determined by measuring the yellow-colored para-nitrophenol released from pNPG at 405 nm.^22^

### HPLC-DAD (High-Performance Liquid Chromatography-Diode Array Detector)

The *Artocarpus heterophyllus* stem bark phenolic sample, at a 10 mg/mL concentration, was injected by means of a model SIL-20A Shimadzu Autosampler. Separations were carried out using a Phenomenex C_18_ column (4.6 mm × 250 mm × 5 µm particle size). The mobile phase was water with 1% phosphoric acid (v/v) (solvent A) and HPLC-grade methanol (solvent B) at a flow rate of 0.6 mL/min and an injection volume of 40 μL. The composition gradient was as follows: 5% solvent B reaching 15% at 10 minutes; 30% solvent B at 35 minutes; 65% solvent B at 50 minutes; and 98% solvent B at 65 minutes; followed by 70 min at isocratic elution until 75 minutes. At 80 minutes the gradient reached the initial conditions again, following the method described by Adefegha et al^23^ with slight modifications. The sample and mobile phases were filtered through a 0.45 µm membrane filter (Millipore) and then degassed by an ultrasonic bath prior to use. Stock solutions of standard references were prepared in methanol at a concentration range of 0.030 to 0.500 mg/mL. Quantifications were carried out by integration of the peaks using the external standard method, at 254 nm for gallic acid, 280 nm for catechin, 327 nm for caffeic acid, and 366 for quercetin and rutin. The chromatography peaks were confirmed by comparing its retention time with those of the reference standards and by DAD spectra (200-600 nm). All chromatography operations were carried out at ambient temperature and in triplicate.

### Induction of Diabetes Mellitus

Single intraperitoneal injection of freshly prepared streptozotocin of 45 mg/kg body weight in citrate buffer (pH 4.5) was used to induce type 2 diabetes mellitus in the Wistar rats. Seventy-two hours after induction, blood samples were obtained from the tips of the rat’s tail and the fasting blood glucose levels were determined using OneTouch Ultra glucometer (LifeScan, USA) to confirm diabetes. Rats with fasting blood glucose levels of ≥200 mg/dL^14^ were used for the experiment.

### Animal Grouping

The rats were divided into 5 groups of 10 animals per group and treated as follows:

*Group A:* nondiabetic control rats received distilled water (Normal control)
*Group B:* untreated diabetic rats received distilled water (Diabetic control)
*Group C:* diabetic rats received 400 mg/kg body weight of free phenol of *A heterophyllus*
*Group D:* diabetic rats received 400 mg/kg body weight of bound phenol of *A heterophyllus*
*Group E:* diabetic rats received 5 mg/kg body weight of metformin

The 400 mg/kg body weight of free and bound phenols were used based on the oral glucose tolerance test carried out by the authors prior to this experiment.

### Collection of Blood Samples

The animals were sacrificed on 28th day of the treatment using the cervical dislocation method, and blood was collected from the jugular vein.

### Preparation of Serum and Tissue Homogenates

Blood samples for serum were collected in plain bottles and allowed to stand at 25°C (room temperature) for 30 minutes to form clots. These were then centrifuged at 3000*g* (gravity) for 5 minutes and the supernatant (serum) was collected with the aid of Pasteur pipettes. The obtained serum was labeled accordingly and stored until further use for various analyses. Additionally, organs of interest such as the liver and pancreas were excised and placed in sterile containers having cold Tris-HCl buffer (pH 7.4). A paper towel was used to dry the organs, which were weighed separately. Thereafter, the organs were homogenized in cold Tris-HCl buffer of 1:10 w/v and centrifuged for 15 minutes at 3000*g* to obtain a clear supernatant.

### Determination of Fasting Blood Glucose

OneTouch Ultra glucometer was used in determining fasting blood glucose levels as described by Ahmad et al.^24^

### Determination of Liver Glycogen

Briefly, 1 g of the excised liver was digested in 1.5 mL of 30% KOH saturated with Na_2_SO_4_ using appropriately labeled test tubes, immersed in ice, and boiled for 30 minutes. Thereafter, 2 mL of 95% ethanol was added to each sample and then centrifuged for 30 minutes at 840*g* twice, for proper precipitation of the glycogen content in samples. Then the supernatant was aspirated, and the precipitate was dissolved in 3 mL of distilled H_2_O. Also, 1 mL of 5% phenol was added to the dissolved glycogen and 5 mL of concentrated H_2_SO_4_ was carefully added. The solution was mixed thoroughly, boiled for 20 minutes, cooled, and the absorbance was read at 600 nm. The glycogen content of the samples was extrapolated from a standard curve and reported as mg/g liver tissue as described by Lo et al.^25^

### Determination of Serum Insulin, Anti-Inflammatory, Glucose Transporter 2, and Homeostatic Model Assessment Score

This was assayed by enzyme-linked immunosorbent assay. The serum insulin, pancreatic IL-6, TNF-α, NF-κB, and hepatic GLUT 2 concentrations were measured by an enzyme-linked immunosorbent assay method.^26^ Homeostatic model assessment (HOMA-IR and HOMA-β) scores were calculated at the end of the intervention according to the following formulas:

HOMA-IR = [(Fasting serum insulin in U/L× Fasting blood glucose in mmol/L)/22.5]

HOMA-β= [(Fasting serum insulin in U/L×20/Fasting blood glucose in mmol/L)-3.5

Note: Conversion factor: insulin (1 U/L = 7.174 pmol/L).

### Determination of Lipid Peroxidation

This was measured as malondialdehyde (MDA) by using the method described by Varshney and Kale.^27^ Briefly, a 0.4 mL aliquot of the liver homogenate was mixed with 1.6 mL of Tris-KCl buffer and 0.5 mL of 30% TCA. Thereafter, 0.5 mL of 0.75% thiobarbituric acid was added to the mixture and placed in a water bath for 45 minutes at 80°C. This was then cooled and centrifuged at 3000*g* for 5 minutes. The clear supernatant was collected and the absorbance was measured against a distilled water blank reference at 532 nm.

### Determination of Antioxidant Enzyme Activities

The activities of catalase (CAT), superoxide dismutase (SOD), and glutathione peroxidase (GPx) were determined as described in commercial kits (Randox Laboratories Ltd, Antrim, UK).

### Determination of Hexokinase

The test tubes were appropriately labeled as blank and test. Two milliliters of 0.2 M Tris buffer, 0.2 mL of 0.09 g/mL glucose, 0.1 mL of 10 mM adenosine triphosphate (ATP), and 0.3 mL of 10 mM magnesium chloride (MgCl_2_) were added to the blank and test, after which 0.1 mL of the sample was added to the test and 0.1 mL of distilled water was added to the blank. The mixture was thoroughly mixed and incubated at 30°C for 15 minutes. Thereafter, 0.5 mL of 5% TCA was added to both blank and test and the absorbance was read using a spectrophotometer at 340 nm.^28^

### Determination of Glucose-6-Phosphatase

This was determined as described in a commercial kit (Randox Laboratories Ltd, Antrim, UK).

### Data Analysis

All data in this study were expressed as the mean ± SEM of 10 replicates unless stated otherwise. Analysis of variance (ANOVA) followed by Tukey-Kramer tests for differences between means was used to detect any significant differences between the treatment groups in the study. This was performed using SPSS version 20.0, and the differences were considered statistically significant at *P* < .05.

## Results

### Polyphenolic-Rich Extract of A heterophyllus on Ferric Reducing Antioxidant Potential

**Figure 1.**
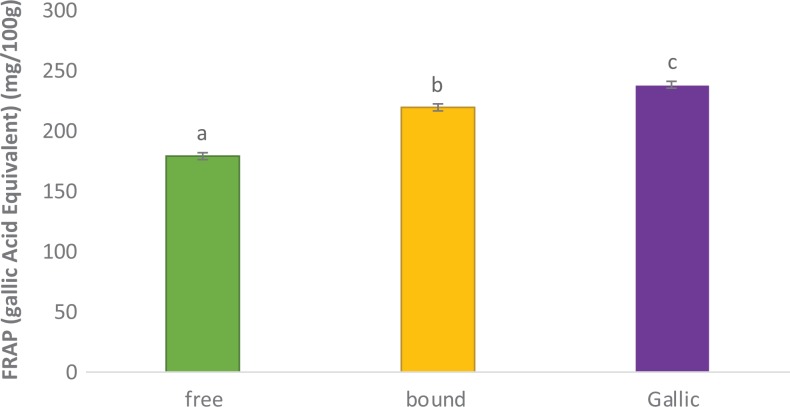
FRAP scavenging ability of polyphenolic-rich extract of *Artocarpus heterophyllus* stem bark. Values are represented as mean ± standard error of mean of triplicate experiments. Bars with different superscripts “a” to “c” are significantly different at *P* < .05.

FRAP scavenging ability of the polyphenolic-rich extract of *A heterophyllus* is depicted in Figure 1. Both free and bound phenolics demonstrated good FRAP with bound phenol having better FRAP scavenging ability than free phenol. Both samples competed favorably with the standard (gallic acid).

### Polyphenolic-Rich Extract of A heterophyllus on DPPH Scavenging Ability

**Figure 2.**
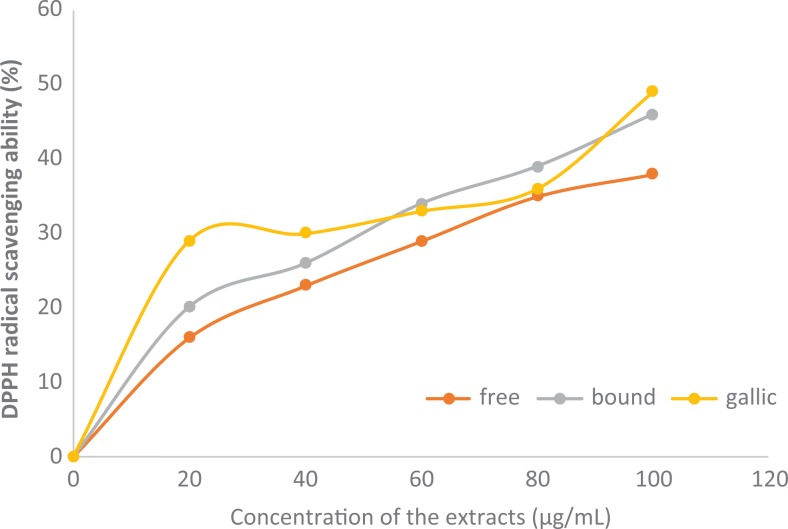
DPPH radical scavenging ability of polyphenolic-rich extract of *Artocarpus heterophyllus* stem bark. Values are represented as mean ± standard error of mean of triplicate experiments.

As the concentration of the polyphenolic-rich extract of *A heterophyllus* stem bark increases so also the DPPH radical scavenging ability of the extract increases (Figure 2). The scavenging ability of bound phenol was significantly (*P* < .05) higher than that of free phenol. Likewise, the gallic acid (standard) shows a significant (*P* < .05) increase in a dose-dependent manner, more than bound phenol.

### Polyphenolic-Rich Extract of A heterophyllus on Hydrogen Peroxide Scavenging Ability

**Figure 3.**
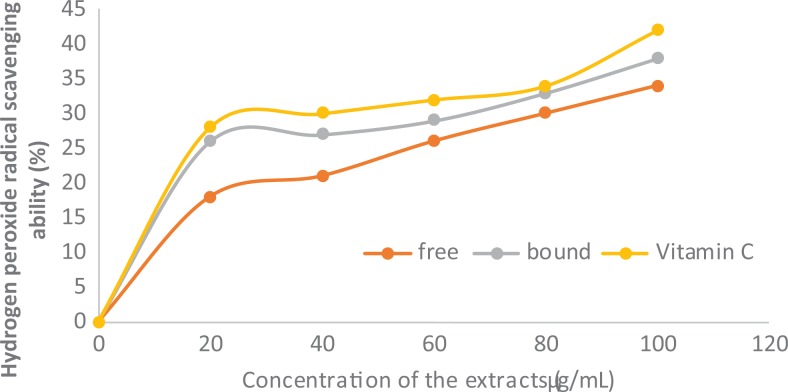
Hydrogen peroxide radical scavenging ability of polyphenolic-rich extract of *Artocarpus heterophyllus* stem bark. Values are represented as mean ± standard error of mean of triplicate experiments.

The hydrogen peroxide scavenging ability of the polyphenolic-rich extract of *A heterophyllus* stem bark also increases in a dose-dependent manner, with bound phenol demonstrating significant (*P* < .05) increase in scavenging ability than free phenol. However, vitamin C (the standard used) was able to scavenge hydrogen peroxide radical more than bound and free phenolics (Figure 3).

### Inhibitory Effects of Polyphenolic-Rich Extract of A heterophyllus Stem Bark Against α-Amylase and α-Glucosidase

**Figure 4.**
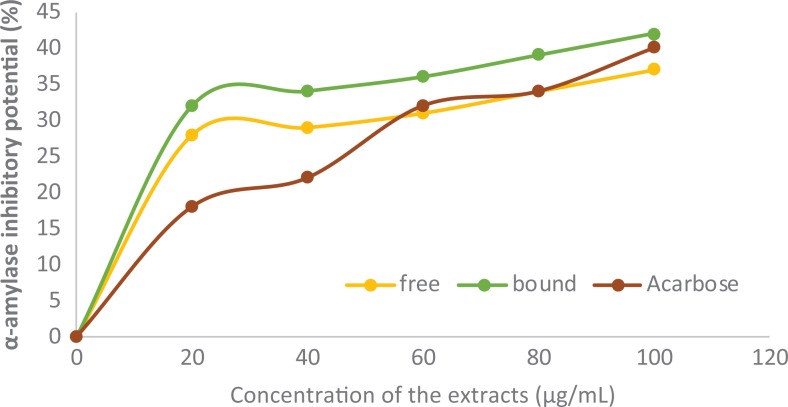
Inhibition of in vitro α-amylase activities by polyphenolic-rich extract of *Artocarpus heterophyllus* stem bark. Values are represented as mean ± standard error of mean of triplicate experiments.

**Figure 5.**
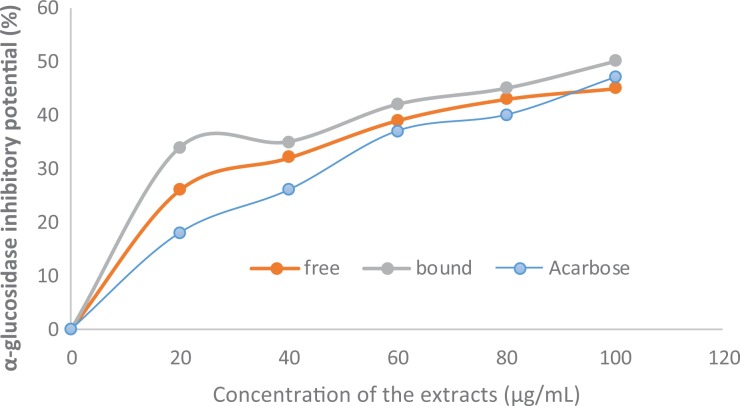
Inhibition of in vitro α-glucosidase activities by polyphenolic-rich extract of *Artocarpus heterophyllus* stem bark.

Figures 4 and 5 show the inhibitory effects of the polyphenolic-rich extract of *A heterophyllus* stem bark against in vitro α-amylase and α-glucosidase. There were significant (*P* < .05) increases in the inhibitory effects of both free and bound phenol against α-amylase and α-glucosidase in a concentration-dependent manner. In addition, bound phenol had significantly (*P* < .05) higher inhibitory activities against both α-amylase and α-glucosidase when compared with free phenol. Furthermore, the bound phenol demonstrated higher inhibitory effects against α-amylase and α-glucosidase than the standard acarbose.

### HPLC Profile of Polyphenolic-Rich Extract of A heterophyllus

**Figure 6.**
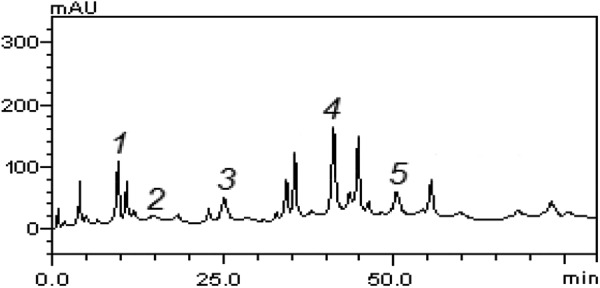
Representative high-performance liquid chromatography profile of *Artocarpus heterophyllus* stem bark. Gallic acid (peak 1), catechin (peak 2), caffeic acid (peak 3), rutin (peak 4), and quercetin (peak 5).

The HPLC profile of the *A heterophyllus* extract was also acquired, as shown in Figure 6. The extract contains the following compounds: gallic acid (retention time [*t*_R_] = 9.71 minutes; peak 1; 2.83 mg/g), catechin (*t*_R_ = 19.05 minutes; peak 2; 0.26 mg/g), caffeic acid (*t*_R_ = 24.93 minutes; peak 3; 1.57 mg/g), rutin (*t*_R_ = 40.68 minutes; peak 4; 4.27 mg/g), and quercetin (*t*_R_ = 50.12 minutes; peak 5; 1.69 mg/g).

### Polyphenolic-Rich Extract of A heterophyllus on Fasting Blood Glucose Levels

**Figure 7.**
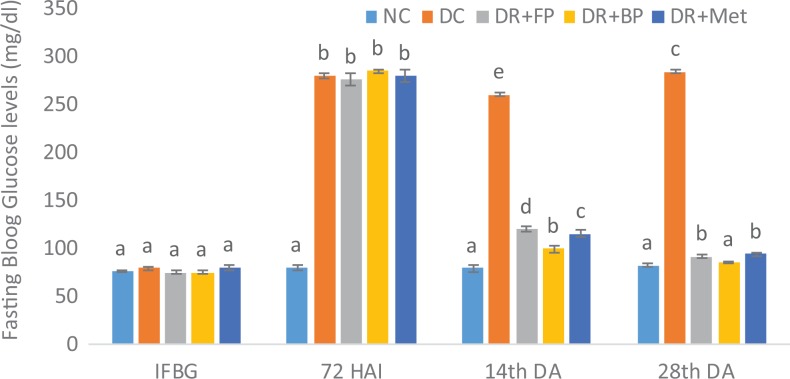
Administration of polyphenols from *Artocarpus heterophyllus* stem bark on fasting blood glucose (FBG) level (mg/dL) of normal and streptozotocin-diabetic rats. Each value is a mean of 10 determinations ± standard error of mean. Bars with different superscripts “a” to “e” are significantly different at *P* < .05. Abbreviations: NC, normal control; DC, diabetic control; DR+FP, diabetic rats administered 400 mg/kg free phenol; DR+BP, diabetic rats administered 400 mg/kg bound phenol; DR+Met, diabetic rats administered 5 mg/kg metformin; IFBG, initial fasting blood glucose; 72 HAI, 72 hours after induction; 14th DA, 14th day of administration; 28th DA, 28th day of administration.

As shown in Figure 7, 72 hours after diabetes induction, fasting blood glucose levels in all the induced groups were significantly (*P* < .05) increased compared with the normal control. On day 14 of the experiment, the diabetic rats administered both free and bound *A heterophyllus* stem bark demonstrated significant (*P* < .05) decrease in fasting blood glucose level compared with diabetic untreated rats. Similarly, on day 28th of the treatment, there was a significant (*P* < .05) reduction in fasting blood glucose levels of diabetic rats administered 400 mg/kg free phenol and 400 mg/kg bound phenol, respectively, as well as those administered 5 mg/kg metformin. However, there was no significant (*P* > .05) increase in fasting blood glucose levels of diabetic rats administered 400 mg/kg bound phenol and normal control rats, or in diabetic rats administered 400 mg/kg free phenol and 5 mg/kg metformin.

### Polyphenolic-Rich Extract of A heterophyllus on Body Weight

On the 28th day of the treatment, there was no significant (*P* > .05) difference in final body weight of diabetic rats administered 400 mg/kg body weight of polyphenolic-rich extract of *A heterophyllus* when compared with normal rats. However, there was a significant (*P* < .05) reduction in the body weight of diabetic control animals when compared with treatment groups (Table 1).

**Table 1.** Administration of Polyphenols From *Artocarpus heterophyllus* Stem Bark on the Body Weight (g) of Normal and Streptozotocin-Diabetic Rats*.

Groups	Initial Body Weight	Final Body Weight
Normal control	159.17 ± 5.04^a^	195.44 ± 4.21^a^
Diabetic control	167.48 ± 4.11^a^	120.67 ± 6.10^c^
Diabetic rats administered 400 mg/kg free phenol	169.92 ± 4.41^a^	192.97 ± 5.24^a^
Diabetic rats administered 400 mg/kg bound phenol	163.16 ± 5.22^a^	194.27 ± 4.45^a^
Diabetic rats administered 5 mg/kg metformin	169.39 ± 4.52^a^	176.10 ± 3.23^b^

* Each value is a mean of 10 determination ± standard error of mean. Values with different superscripts “a” to “c” across the column are significantly different at *P* < .05.

### Polyphenolic-Rich Extract of A heterophyllus on Liver Glycogen

**Figure 8.**
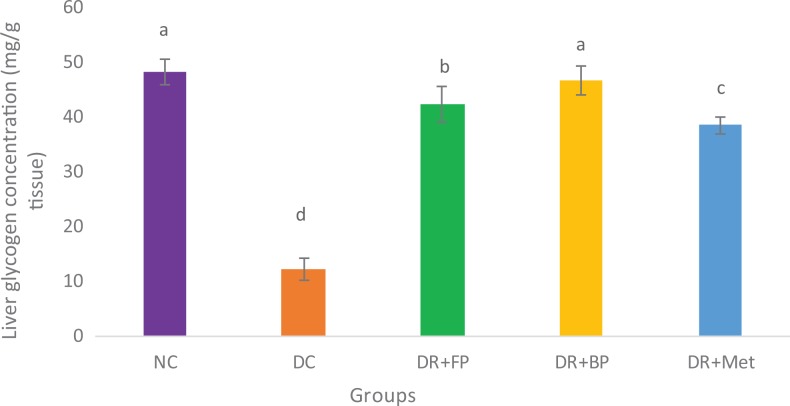
Administration of polyphenols from *Artocarpus heterophyllus* stem bark on liver glycogen concentration (mg/g tissue) of normal and streptozotocin-diabetic rats. Each value is a mean of 10 determinations ± standard error of mean. Bars with different superscripts “a” to “d” are significantly different at *P* < .05 Abbreviations: NC, normal control; DC, diabetic control; DR+FP, diabetic rats administered 400 mg/kg free phenol; DR+BP, diabetic rats administered 400 mg/kg bound phenol; DR+Met, diabetic rats administered 5 mg/kg metformin.

Glycogen concentration significantly (*P* < .05) decreased in diabetic control rats compared with the normal control, as well as in diabetic rats administered both free and bound phenolic extracts and in diabetic rats administered metformin (Figure 8). Nevertheless, at day 28 of the experiment, there was no significant (*P* < .05) increase in both diabetic rats administered 400 mg/kg bound phenol and normal control rats, while there was a significant (*P* < .05) increase in liver glycogen concentration of diabetic rats administered 400 mg/kg free phenol compared with diabetic rats administered metformin.

### Polyphenolic-Rich Extract of A heterophyllus on Insulin, HOMA-IR, and HOMA-β

Table 2 shows the effect of polyphenolic-rich extract of *A heterophyllus* stem bark in streptozotocin-induced diabetic rats on insulin concentration, HOMA-IR, and HOMA-β. There was no significant (*P* > .05) increase in insulin concentration of the normal control and in diabetic rats administered 400 mg/kg free and bound phenol. A significant (*P* < .05) decrease was observed in the insulin of the diabetic control group compared with the normal control group as well as in diabetic rats administered extracts and metformin. There was significant (*P* < .05) elevation in the HOMA-IR of the diabetic control rats when compared with other groups, whereas the reverse was the case in HOMA-β.

### Polyphenolic-Rich Extract of A heterophyllus on Oxidative Stress Biomarkers

There was a significant (*P* < .05) increase in the concentration of MDA in the liver of diabetic control rats when compared with other groups (Table 3). However, diabetic rats administered polyphenols of *A heterophyllus* stem bark extracts demonstrated significant (*P* < .05) reduction in MDA concentration, with no significant (*P* > .05) difference in normal control and in diabetic rats administered 400 mg/kg bound phenol and 5 mg/kg metformin. Meanwhile, the antioxidant enzyme activities of SOD, CAT, and GPx in the liver of streptozotocin-induced diabetic rats are likewise depicted in Table 2. The diabetic control rats showed significant (*P* < .05) reduction in SOD, CAT, and GPx activities in the liver compared with diabetic rats administered polyphenols of *A heterophyllus* stem bark extracts and metformin.

**Table 2.** Administration of Polyphenols From *Artocarpus heterophyllus* Stem Bark on Insulin Concentration, HOMA-IR, and HOMA-β of Normal and Streptozotocin-Diabetic Rats*.

Groups	Insulin (pmol/L)	HOMA-IR	HOMA-β
Normal control	100.21 ± 1.01^a^	2.64 ± 0.11^a^	240.56 ± 2.31^a^
Diabetic control	26.14 ± 2.11^c^	3.13 ± 0.21^d^	5.53 ± 1.15^e^
Diabetic rats administered 400 mg/kg free phenol	94.12 ± 4.11^a^	2.77 ± 0.20^d^	151.55 ± 3.01^c^
Diabetic rats administered 400 mg/kg bound phenol	98.46 ± 3.12^a^	2.69 ± 0.31^c^	200.01 ± 4.01^b^
Diabetic rats administered 5 mg/kg metformin	92.31 ± 2.01^b^	2.77 ± 0.01^b^	139.88 ± 2.45^d^

* Each value is a mean of 10 determination ± standard error of mean. Values with different superscripts “a” to “e” across the column are significantly different at *P* < .05.

**Table 3.** Administration of Polyphenols From *Artocarpus heterophyllus* Stem Bark on Oxidative Stress Biomarker (U/mg Protein) of Normal and Streptozotocin-Diabetic Rats*.

Groups	SOD	CAT	GPx	MDA
Normal control	86.21 ± 2.06^a^	64.27 ± 2.11^a^	96.23 ± 3.26^a^	4.09 ± 2.18^a^
Diabetic control	20.46 ± 1.06^e^	15.69 ± 1.10^e^	27.87 ± 2.13^e^	12.19 ± 1.16^c^
Diabetic rats administered 400 mg/kg free phenol	76.21 ± 4.10^c^	52.46 ± 2.18^c^	82.34 ± 3.12^c^	5.02 ± 2.04^b^
Diabetic rats administered 400 mg/kg bound phenol	79.21 ± 3.14^b^	59.68 ± 3.10^b^	86.12 ± 1.29^b^	4.60 ± 1.24^a^
Diabetic rats administered 5mg/kg metformin	60.96 ± 2.32^d^	46.47 ± 3.20^d^	73.21 ± 3.16^d^	4.56 ± 1.36^a^

Abbreviations: SOD, superoxide dismutase; CAT, catalase; GPx, glutathione peroxidase; MDA, malondialdehyde.

* Each value is a mean of 10 determination ± standard error of mean. Values with different superscripts “a” to “e” across the column are significantly different at *P* < .05.

### Polyphenolic-Rich Extract of A heterophyllus on Some Carbohydrate Metabolism Enzymes

**Figure 9.**
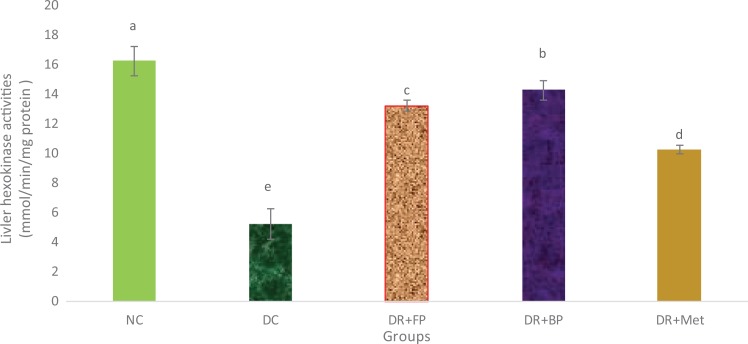
Administration of polyphenols from *Artocarpus heterophyllus* stem bark on liver hexokinase activity of normal rats. Each value is a mean of 10 determinations ± standard error of mean. Bars with different superscripts “a” to “e” are significantly different at *P* < .05. Abbreviations: NC, normal control; DC, diabetic control; DR+FP, diabetic rats administered 400 mg/kg free phenol; DR+BP, diabetic rats administered 400 mg/kg bound phenol; DR+Met, diabetic rats administered 5 mg/kg metformin.

**Figure 10.**
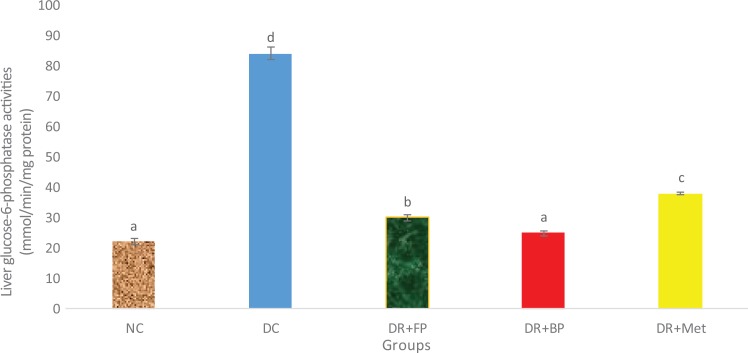
Administration of polyphenols from *Artocarpus heterophyllus* stem bark on liver glucose-6-phosphatase activity of normal and streptozotocin-diabetic rats. Each value is a mean of 10 determinations ± standard error of mean. Bars with different superscripts “a” to “d” are significantly different at *P* < .05. Abbreviations: NC, normal control; DC, diabetic control; DR+FP, diabetic rats administered 400 mg/kg free phenol; DR+BP, diabetic rats administered 400 mg/kg bound phenol; DR+Met, diabetic rats administered 5 mg/kg metformin.

Figures 9 and 10 show the administration of polyphenols of the *A heterophyllus* stem bark on the activities of some carbohydrate metabolic enzymes. The hexokinase activity of the diabetic control rats compared with diabetic rats administered phenolic extracts (both bound and free) and metformin was significantly (*P* < .05) reduced. Diabetic rats administered bound and free phenols showed more significant (*P* < .05) or greater hexokinase activity than diabetic rats administered metformin. Whereas the activity of glucose-6-phosphatase was significantly (*P* < .05) increased in diabetic control rats compared with diabetic rats administered phenolic extracts and metformin, diabetic rats administered 400 mg/kg bound phenol and normal control rats demonstrated no significant (*P* > .05) increase.

### Polyphenolic-Rich Extract of A heterophyllus on Glucose Transporter 2

**Figure 11.**
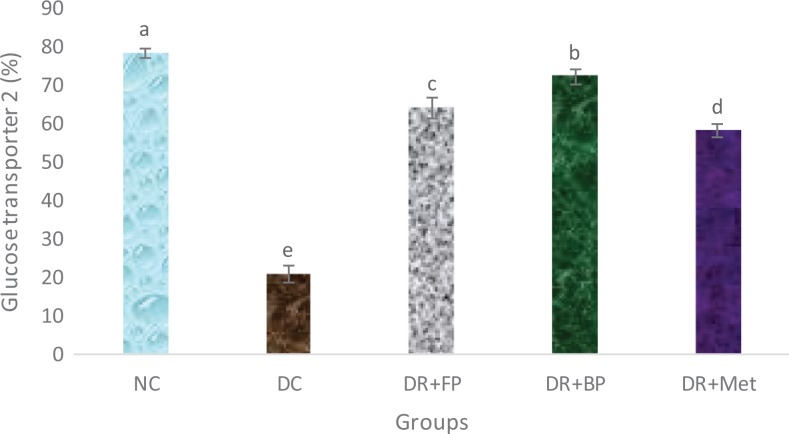
Administration of polyphenols from *Artocarpus heterophyllus* stem bark on hepatic GLUT 2 concentration of normal and streptozotocin-diabetic rats. Each value is a mean of 10 determinations ± standard error of mean. Bars with different superscripts “a” to “e” are significantly different at *P* < .05. Abbreviations: NC, normal control; DC, diabetic control; DR+FP, diabetic rats administered 400 mg/kg free phenol; DR+BP, diabetic rats administered 400 mg/kg bound phenol; DR+Met, diabetic rats administered 5 mg/kg metformin.

Figure 11 depicts the concentration of glucose transporter 2 (GLUT 2), which was significantly (*P* < .05) decreased in the diabetic control rats than in the diabetic rats administered phenolic extracts and metformin. Additionally, the diabetic rats administered 400 mg/kg free and bound phenols demonstrated significant (*P* < .05) increase in GLUT 2 concentration than in the diabetic rats administered metformin.

### Polyphenolic-Rich Extract of A heterophyllus on Some Anti-Inflammatory

**Figure 12.**
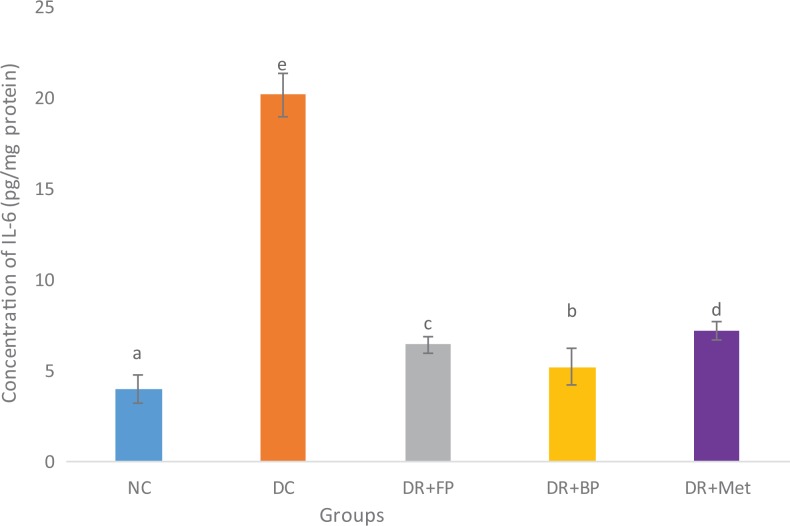
Administration of polyphenols from *Artocarpus heterophyllus* stem bark on pancreatic interleukin (IL)-6 concentration of normal and streptozotocin-diabetic rats. Each value is a mean of 10 determinations ± standard error of mean. Bars with different superscripts “a” to “e” are significantly different at *P* < .05. Abbreviations: NC, normal control; DC, diabetic control; DR+FP, diabetic rats administered 400 mg/kg free phenol; DR+BP, diabetic rats administered 400 mg/kg bound phenol; DR+Met, diabetic rats administered 5 mg/kg metformin.

**Figure 13. fig13-2515690X20916123:**
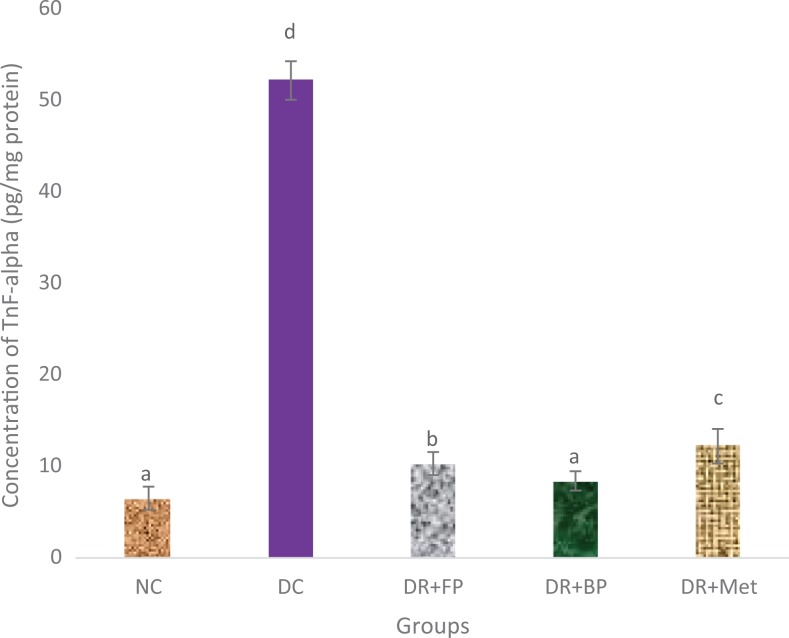
Administration of polyphenols from *Artocarpus heterophyllus* stem bark on pancreatic tumor necrosis factor (TNF)-α concentration of normal and streptozotocin-diabetic rats. Each value is a mean of 10 determinations ± standard error of mean. Bars with different superscripts “a” to “d” are significantly different at *P* < .05. Abbreviations: NC, normal control; DC, diabetic control; DR+FP, diabetic rats administered 400 mg/kg free phenol; DR+BP, diabetic rats administered 400 mg/kg bound phenol; DR+Met, diabetic rats administered 5 mg/kg metformin.

There was a significant (*P* < .05) increase in anti-inflammatory (IL-6, TNF-α, and NF-κB) levels of pancreatic diabetic control rats compared with diabetic rats administered the polyphenolic-rich extract of *A heterophyllus* stem bark in streptozotocin-induced diabetic rats (Figures 12 [Fig fig13-2515690X20916123]–14). Diabetic rats administered phenolic extracts demonstrated a significant (*P* < .05) decrease in all the anti-inflammatory effect studied compared with the diabetic rats administered metformin.

**Figure 14.**
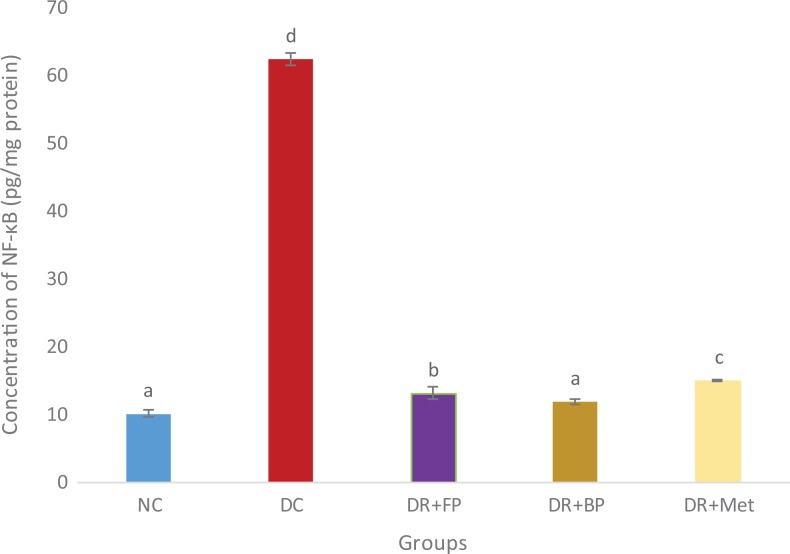
Administration of polyphenols from *Artocarpus heterophyllus* stem bark on pancreatic NF-κB concentration of normal and streptozotocin-diabetic rats. Each value is a mean of 10 determinations ± standard error of mean. Bars with different superscripts “a” to “d” are significantly different at *P* < .05. Abbreviations: NC, normal control; DC, diabetic control; DR+FP, diabetic rats administered 400 mg/kg free phenol; DR+BP, diabetic rats administered 400 mg/kg bound phenol; DR+Met, diabetic rats administered 5 mg/kg metformin.

## Discussion

In the present study, polyphenolic-rich extract of *A heterophyllus* stem bark demonstrated strong antidiabetic properties. This is in accordance with earlier reports on the use of medicinal plants in the management of diabetes mellitus as documented by Koehn and Carter.^29^ Medicinal plants with free radical scavenging abilities as well as α-amylase and α-glucosidase inhibitory activities have especially been reported to be useful in managing diabetes mellitus. In this study, a polyphenol-rich extract of *A heterophyllus* stem bark demonstrated abilities to scavenge free radicals that easily accept electrons or hydrogen radicals to become stable diamagnetic molecules, and this finding is in accordance with a report by Ajiboye et al.^14^ This, therefore, hints that this extract may be useful in managing diseases that result from the accumulation of oxidative stress, with diabetes mellitus being such an example.

Inhibiting carbohydrate hydrolyzing enzymes, particularly α-amylase and α-glucosidase in the gastrointestinal tract, plays an important role in minimizing postprandial hyperglycemia.^30^ In this study, the extract demonstrates the ability to inhibit these enzymes in a concentration-dependent manner, probably due to gallic acid, catechin, caffeic acid, rutin, and quercetin present in the extract. These compounds are well admitted as potential antioxidants, free radical scavengers, and inhibitors of lipid peroxidation among others.^30– 32^ The polyphenolic-rich extract of *A heterophyllus* stem bark shows a significant increase in the inhibitory properties of α-amylase and α-glucosidase than acarbose, suggesting that it will be useful in curtailing the side effects related to synthetic drugs.

Insulin deficiency in diabetes mellitus patients encourage gluconeogenesis,^8^ which may be attributed to a reduction in body weight of diabetic rats as observed in this study. However, the administration of polyphenolic-rich extract of *A heterophyllus* stem bark was able to reverse this abnormal decrease in the body weight of the diabetic rats. This may be linked to the ability of the extract to increase insulin concentration associated with the phenolic compounds in the extract.

The balance between insulin and glucagon to maintain a stable blood glucose level, called glucose homeostasis, is crucial for the utilization of glucose by the liver, muscles, and adipose tissues. In this study, streptozotocin was used to induce hyperglycemia in rats because it is a methylating agent for DNA and acts in damaging the β-cells of the pancreas.^33^ One of the main features of diabetes mellitus is excessive glucose concentration (hyperglycemia) caused by insulin deficiency. Persistent hyperglycemia over time may affect almost all the organs in the body system, especially the brain, retina, kidney, and liver.^34– 36^ It was observed in this study that diabetic rats administered the polyphenolic-rich extract of *A heterophyllus* stem bark was effective in reducing blood glucose and compared favorably to the normal control and metformin administered rats. This implies that the polyphenolic-rich extract of *A heterophyllus* stem bark is able to increase the concentration of insulin resulting in a lower glucose level (normoglycemia), suggesting an antidiabetic activity, which is in consonance with the report of Ajiboye et al.^14^ Moreover, the normoglycemia observed in the diabetic rats administered polyphenolic-rich extract might also be due to an increase in glycogenesis, inhibition of gluconeogenesis in the liver, or inhibition of absorption of glucose from the intestine.^36^

Diabetes mellitus has been characterized by a decrease in glycogen storage due to defects in insulin secretions, which comes as a result of selective destruction of β-cells of the pancreas.^8^ Malini et al^37^ reported that glycogen is the primary intracellular storage form of glucose and its levels in various tissues are a direct reflection of insulin concentration, since insulin promotes intracellular glycogenesis by stimulating glycogen synthase and inhibiting glycogen phosphorylase. In this study, glycogen storage was impaired in diabetic control rats. Administration of the polyphenolic-rich extract to diabetic rats significantly increased the level of hepatic glycogen levels and this may be attributed to the ability of the extract to increase insulin concentration.

One of the main problems with diabetes mellitus patients is deficient insulin secretion, which is responsible for the various complications seen in such patients.^8^ Insulin is a product of enzymatic cleavage of pro-insulin, which is secreted into the blood circulatory system. Insulin concentration was significantly decreased in diabetic control rats, due to the selective destruction of β-cells of the pancreas. But at the end of 28 days of oral administration of polyphenolic-rich extract of *A heterophyllus* stem bark to diabetic rats, significantly increased insulin concentrations. This may be attributed to the bioactive compounds present in the extract. These compounds have the ability to regenerate the damaged β-cells of the pancreas and boost insulin secretion. This was supported by the significant increase in HOMA-β (β-cell function) in both polyphenolic-rich extract and metformin-treated groups compared with the diabetic control group. Additionally, a significant decrease in the HOMA-IR index in diabetic rats administered polyphenolic-rich extract and metformin compared with the diabetic control rats support the improvement in insulin sensitivity and secretion as well as in stimulation of peripheral glucose absorption in those groups. Furthermore, this claim may be attributed to the antioxidative ability of the bioactive compounds found in the extract, which is in accordance with the report of Ilic et al^38^ on *Aframomum melegueta* Schum.

Patel et al^39^ reported that an increase in ROS production may trigger damage to fundamental biomolecules like proteins, lipids, carbohydrates, and DNA, thereby leading to the incapability of the body’s defense mechanism in protecting cellular integrity. Free radical accumulation causes lipid peroxidation, which is a process by which the lipids of the cell membrane undergo catabolism, leading to tissue damage. The polyunsaturated fatty acids of the liver are compromised by the broken-down cell membrane structure, leading to disruption in its functionality.^40^ In this study, MDA levels (a marker for lipid peroxidation) increased significantly in the liver of diabetic rats when compared with normal control. This increase in MDA levels observed in the diabetic rats suggests damage to cell membrane lipids that can lead to an increase in ROS generation.^41^ Administration of diabetic rats with polyphenolic-rich extract of the *A heterophyllus* stem bark led to a significant decrease in the levels of MDA by reducing lipid peroxidation. This may be attributed to the antilipid peroxidation of the bioactive compounds present in the extract.

In another vein, the activities of antioxidant enzymes (SOD, CAT, and GPx) in diabetic mellitus patients normally reduce due to amelioration of ROS-induced oxidative stress as reported by Naugler and Karin.^42^ The first line of defense against ROS is SOD, because it is responsible for the dismutation of superoxide radicals to water, while catalase eliminates hydrogen peroxide and GPx uses glutathione as a substrate to detoxify hydrogen and lipid peroxides.^9,43^ There was an observed reduction in the above-mentioned antioxidant enzyme activities in the diabetic control rats when compared with other diabetic rats. However, at the end of the experiment, diabetic rats administered the polyphenolic-rich extract demonstrated a significant increase in these enzyme activities, which may be attributed to the antioxidative nature of the bioactive compounds present in the extract.

Insulin insufficiency in diabetic mellitus patients may actually be the main reason responsible for a significant reduction in the activities of liver hexokinase because its activity depends on insulin. Hexokinase is an important regulatory enzyme in the oxidation of glucose in the liver. In this study, the hexokinase activity of diabetic control rats was impaired, which triggered a reduction in glucose oxidation (via glycolysis) and led to hyperglycaemia^8,44^ as observed earlier. However, there was a significant increase in the liver hexokinase activities of diabetic rats that were administered the polyphenolic-rich extract of *A heterophyllus* stem bark, probably due to the regeneration of damaged pancreatic β-cells by the extract, which encouraged an increase in insulin concentration. A decrease in insulin concentration and an increase in glucagon concentration in diabetic mellitus patients are responsible for significant increases in glucose-6-phosphatase activity, an important enzyme in gluconeogenesis and glycogenolysis.^45^ This was observed in the current study, but it was, however, ameliorated after administering the polyphenolic-rich extract to diabetic rats probably due to an increase in insulin concentration.

Glucose transporter 2 (GLUT 2), or solute carrier family 2, facilitates the transport of glucose out of the mucosal cells, thereby allowing its entry into the portal circulation and its transportation to the liver, pancreas, small intestine, and kidney. GLUT 2 functions mainly in the rapid uptake and release of glucose.^46^ Maughana^47^ reported that glucose transport is the rate-limiting step in carbohydrate metabolism which is facilitated by glucose transporters (GLUT 2). In diabetes mellitus patients, the hepatic concentration of GLUT 2 normally decreases,^46^ and this is consistent with results observed in this study. However, after the administration of diabetic rats with the *A heterophyllus* stem bark polyphenolic-rich extract, there was significant increase in GLUT 2 concentration. This may be a pathway to reverse the glucose uptake in liver cells coupled with an increase in insulin secretion as demonstrated by the extract. This can be a turning point in the management of diabetes mellitus.

Persistent hyperglycemia in diabetes mellitus patients may trigger increased inflammation in tissues (especially in pancreatic cells) due to responses to harmful stimuli or damage to cells.^7^ Pancreatic IL-6, TNF-α, and NF-κB cytokines play a crucial role in hyperglycemia-induced diabetic rats.^36,48,49^ Diabetic control rats showed an increased level of IL-6, TNF-α, and NF-κB. Conversely, these were ameliorated after the administration of diabetic rats with polyphenolic-rich extract of *A heterophyllus* stem bark. This demonstrated the anti-inflammatory properties of the bioactive compounds present in the extract.

## Conclusion

From this study, it can be deduced that free and bound phenolic extracts of the *A heterophyllus* stem bark demonstrated high antioxidant potentials, inhibited both α-amylase and α-glucosidase, and possess gallic acid, catechin, caffeic acid, rutin, and quercetin as bioactive compounds in the extract. These extracts ameliorate fasting blood glucose levels, increase liver glycogen, improve insulin concentration, enhance pancreatic β-cell and their functions; improve antioxidant enzymes, liver hexokinase activities, and GLUT 2; and reduce glucose-6-phosphatase activity and improve the concentrations of all the anti-inflammatory parameters determined. The brilliant performance of the extracts may be attributed to its bioactive compounds.
